# Comparative Study Between Intravenous Patient-Controlled Analgesia Morphine and Computerized Ambulatory Delivery Device Epidural Morphine for Post Operative Analgesia for Nuss Procedure in Pectus Excavatum: A Retrospective Analysis

**DOI:** 10.7759/cureus.41301

**Published:** 2023-07-03

**Authors:** Vishal Bhatnagar, Ravi Kumar, Anshu Singh, Manish Singh, L M Darlong, Amit Kumar Mittal

**Affiliations:** 1 Surgical Intensive Care Unit, Rajiv Gandhi Cancer Institute and Research Centre, New Delhi, IND; 2 Plastic Surgery, King George's Medical University, Lucknow, IND; 3 Plastic Surgery, Lala Lajpat Rai Memorial Medical College, Meerut, Meerut, IND; 4 Thoracic Onco-Surgery, Rajiv Gandhi Cancer Institute and Research Centre, New Delhi, IND; 5 Anaesthesia, Rajiv Gandhi Cancer Institute and Research Centre, New Delhi, IND

**Keywords:** epidural, morphine, cadd, pca, nuss procedure

## Abstract

Background: Pectus deformities are commonly seen in chest wall deformities among the pediatric age group. Pectus deformities occur due to defective growth of the sternum and its surrounding cartilage. The Nuss procedure is the technique of choice for correcting the deformity surgically which includes placing a convex bar under the sternum without resection or injury to costal cartilages. Adequate pain control is utmost to improve wound healing, patient satisfaction, short hospital stays, and decrease the financial burden on attendants. Therefore, it is necessary to investigate which analgesic method is more advantageous for the Nuss procedure.

Objective: To compare the analgesic effects of intravenous patient-controlled analgesia (IVPCA) morphine versus computerized ambulatory delivery device (CADD) epidural morphine on acute post-operative pain management in Nuss procedures.

Methods: A retrospective study was done at Rajiv Gandhi Cancer and Research Hospital, New Delhi from 2015 to 2020 to assess the efficacy and safety between IVPCA morphine and CADD epidural for post-operative analgesia following pectus excavatum repair. A total of 34 cases of Nuss procedures were taken with 17 cases in each group. Group 1 (intravenous PCA morphine) was given 39 ml normal saline + 6 ml morphine (total 45 ml, 2 mg/ml morphine), set at demand dose 0.5 ml, i.e. 1 mg, lockout interval 7 minutes, doses per hour was six and Group 2 (CADD epidural morphine) was given 42 ml normal saline + 3 ml morphine (1 mg/ml morphine) with continuous infusion at the rate of 0.5 ml/hr. Demand dose 0, lockout interval nil. Visual analog pain scores using a scale of 0-10 and Ramsay Sedation Score (RSS) scores were obtained on arrival at the post-anesthesia care unit, at 12, 24, 48, and 72 hours throughout the subsequent hospital stay.

Results: This study yielded positive information about our experience with the pectus post-operative pain management. The mean visual analog scale (VAS) score was lower in Group 1 compared to Group 2 but significantly different at 12 and 72 hours only. The mean RSS score was comparable between groups. The mean hospital stay (days) and requirement of rescue analgesia doses were 3.47±0.51 and 0.12±0.33 in Group 1 and 4.76±0.44, 0.59±1.12 in Group 2.

Conclusion: Both IVPCA morphine and CADD morphine were effective in controlling post-surgical pain in the Nuss procedure, but IVPCA morphine was better as compared to CADD morphine in this regard because it was noninvasive, safe, and cost-effective with non-significant complications.

## Introduction

Pectus deformities are one of the most commonly seen chest wall deformities among the pediatric age group and have an occurrence of one in 300 births [1]. Pectus deformities occur due to defective growth of the sternum and its surrounding cartilage. Pectus deformities result in poor self-image and hesitation in social interactions with a psychological impact that may be detrimental for adolescents and young adults, thereby causing a significant reduction in their quality of life. The Nuss technique is the procedure of choice for correcting the deformity surgically which includes placing a convex bar under the sternum without resection or injury to costal cartilages. The following procedure involves the placement of a curved steel bar which is individually shaped for each patient, through a retrosternal tunnel created endoscopically. Once the bar is placed in position, the steel bar is turned over, thereby leading to the correction of deformity [2-6]. Despite being classified as a minimally invasive technique, there is considerable post-operative pain among the patients [7]. Intravenous patient-controlled analgesia (IVPCA) with morphine and continuous epidural analgesia administered by a computerized ambulatory delivery device (CADD epidural morphine), using an opioid alone or in combination with a local anesthetic, are two major advances in managing pain after major surgery [8].

In our study, we used morphine alone. Adequate pain control is utmost to improve wound healing, patient satisfaction, short hospital stays, and decrease the financial burden on the attendant. Although intravenous PCA morphine is known to be more advantageous in controlling pain if patients refuse to place an epidural catheter or those who have coagulopathy and infection at the insertion site [7,8]. The CADD epidural morphine is a pain-relieving procedure that has limitations due to the requirement of placing an epidural catheter due to which patients remain catheterized on the back and chances of infection and complications increase [7,8].

Although the hypothesis is that IVPCA morphine has a better post-operative analgesic effect when compared with CADD morphine. Still, there is conflicting evidence for this. Therefore, it is necessary to investigate which analgesic method is more advantageous for the Nuss procedure. Hence, the primary objective of this study was to compare the analgesic effects of intravenous PCA morphine versus CADD epidural morphine on acute post-operative pain management in the Nuss procedure.

## Materials and methods

Study design and participants

This study was approved by the Institutional Review Board (RGCIRC/IRB-BHR/107/2020) at Rajiv Gandhi Cancer Hospital and Research Center, Rohini, New Delhi. Due to the retrospective observational study design, requirements for informed consent were waived. The pain service database of our hospital was reviewed comprehensively to retrieve the data of all the patients who underwent the Nuss procedure in a period of five years, including the years 2020 through 2015. A total of 34 cases of Nuss procedure were taken with 17 cases in each group.

Inclusion and exclusion criteria

Patients with pectus excavatum who underwent Nuss procedure between 2015-2020. Patients were excluded if pectus excavatum repair was fulfilled through a surgical approach other than the Nuss procedure.

Sample size and data collection

A total of 34 patients were fulfilling the inclusion and exclusion criteria. Among them, 17 had received IVPCA morphine while 17 had received CADD morphine. Being retrospective in nature, no sample size calculation was done.

Two groups were considered for the study. Group 1 (intravenous PCA morphine) was given 39 ml normal saline + 6 ml morphine (total 45 ml) 2mg/ml morphine, set at demand dose 0.5 ml i.e., 1 mg, lockout interval 7 minutes, doses per hour was six. Group 2 (CADD epidural morphine) was given 42 ml normal saline + 3 ml morphine (1 mg/ml morphine) with continuous infusion at 0.5ml/hr. Demand dose = 0, Lockout interval = nil.

The dosing regime was per the intensive care unit (ICU) guidelines of the research institute where the study was conducted. Local anesthetics were not used as per the SOPs (standard operating procedure) of the ICU of the study hospital. Low-dose opioids were used to minimize the various complications of opioids, especially, respiratory depression.

Both groups were matched at baseline for age, gender, and weight. However, there was a significant difference in the height and BMI of patients in both the groups at baseline which was due to the retrospective nature of this study. Variables were analyzed over the initial 72‑h post-operative period including pain scores (VAS), Ramsay Sedation Score (RSS), the incidence of various complications (nausea, vomiting, etc.), hospital stay, and frequency of rescue analgesia (injection tramadol 50 mg iv SOS). Visual analog pain scores (VAS) using a scale of 0-10 and RSS scores were obtained on arrival at the post-anesthesia care unit (PACU), at 12 hours, 24 hours, 48 hours, and 72 hours throughout the subsequent hospital stay. VAS is one of the pain rating scales used in epidemiologic and clinical research to measure the intensity or frequency of various symptoms. RSS score divides a patient's level of sedation into six categories ranging from severe agitation to deep coma. In the post-operative period, we had seen the hospital stay, doses of rescue analgesia, and complications.

Outcome measures

The primary objective of this study was to compare the post-operative analgesic effect (VAS and frequency of rescue analgesia) between patients receiving intravenous PCA morphine and epidural CADD morphine. The secondary outcome was to assess the safety and efficacy of drugs administered through different routes. The thoracic epidural catheter was placed after induction using sterile techniques and confirmation of catheter placement was done by midline loss of resistance at the T5, T6, or T7 level. It was not ultrasonography (USG)-guided. Thoracic epidurals were dosed with CADD morphine. Efficacy was determined by the post-operative analgesic effect and sedation while safety was assessed by the complications and hospital stay.

Statistical analysis

SPSS version 21.0 (IBM Corp., Armonk, NY) was used for statistical analysis. Data was presented as mean (standard deviation) and percentage (%). The Chi-square test was used to compare the dichotomous/categorical variables. The unpaired Student's t-test was used to compare discrete variables between Group 1 and Group 2. A value of P < 0.05 was considered statistically significant.

## Results

Table 1 shows the baseline characteristics of the patients. The mean age of 17 patients in Groups 1 and 2 was 23.59±6.18 (years) and 21.65±3.82 (years) respectively. The percentage of males and females was 88.24% and 11.76% in Group 1 and 76.47% and 23.53% in Group 2. The mean weight (Kg), height (cm), and BMI (Kg/m^2^) were 63.88±11.73, 172.82±10.49 and 21.31±2.91 in Group 1 and 62.65±7.185, 162.24±5.44, and 23.75±1.93 in Group 2. The mean age weight and gender were not significantly different between groups. The mean height and BMI were significantly different between groups.

**Table 1:** Baseline characteristics of the study participants

	Group 1 (n=17)		Group 2 (n=17)		p-Value
	Mean/n	±SD/%	Mean/n	±SD/%	
Age (years)	23.59	6.18	21.65	3.82	0.279
Gender					
Male	15	88.24%	13	76.47%	0.653
Female	2	11.76%	4	23.53%	
Weight (kg)	63.88	11.73	62.65	7.185	0.714
Height (cm)	172.82	10.49	162.24	5.44	0.001^*^
BMI (kg/m2)	21.31	2.91	23.75	1.93	0.007^*^

Table 2 shows the mean VAS score and RSS score comparison between Group 1 and Group 2 at PACU, 12 hours, 24 hours, 48 hours, and 72 hours. The mean VAS score was lower in Group 1 as compared to Group 2 but significantly different at 24 and 72 hours only. The mean RSS score was comparable between groups.

**Table 2:** Comparisons of VAS and RSS scores *=Significant (p<0.05) Comparisons of VAS and RSS scores between Group 1 and Group 2 at PACU, 12, 24, 48, and 72 hours VAS: Visual analog scale; RSS: Ramsay sedation score; PACU:

		Group 1 (n=17)		Group 2 (n=17)		p-Value
		mean	±SD	mean	±SD	
VAS	PACU	6.24	0.83	6.65	0.49	0.089
	12 HRS	1.29	1.69	2.41	1.50	0.050
	24 HR	0.82	0.39	2.12	1.62	0.003^*^
	48 HR	0.94	0.24	1.00	0.00	0.325
	72 HR	0.65	0.49	1.00	0.00	0.006^*^
RSS	PACU	1.00	0.00	1.00	0.00	-
	12 HRS	2.00	0.00	2.00	0.00	-
	24 HR	2.00	0.00	2.00	0.00	-
	48 HR	2.00	0.00	2.00	0.00	-
	72 HR	2.00	0.00	2.00	0.00	-

Table 3 shows the hospital stay (days), the requirement of rescue analgesia doses, and receiving additional PCA bolus doses. The mean hospital stay (days) and requirement of rescue analgesia doses were 3.47±0.51 and 0.12±0.33 in Group 1 and 4.76±0.44, 0.59±1.12 in Group 2. The mean hospital stay (days) and requirement of rescue analgesia doses were lower in Group 1 compared to Group 2 but hospital stay (days) were significantly different.

**Table 3:** Hospital stay (days) and requirement of rescue analgesia doses in both groups

	Group 1 (n=17)		Group 2 (n=17)		p-Value
	mean	±SD	mean	±SD	
Hospital Stay (Days)	3.47	0.51	4.76	0.44	<0.001^*^
Requirement of rescue analgesia doses	0.12	0.33	0.59	1.12	0.107

Table 4 shows the comparisons of complications in Group 1 and Group 2. The percentages of nausea, vomiting, itching, and constipation were 5.88%, 0.00%,0.00%, and 0.00%, Group 1 and 17.65%,0.00%,17.65%, and 11.76%, Group 2, respectively. The percentage of complications was not significantly different in Group 1 and Group 2.

**Table 4:** Comparison of complications between Groups 1 and 2

	Group 1 (n=17)		Group 2 (n=17)		p-Value
	n	%	n	%	
Nausea	1	5.88	3	17.65	0.595
Vomiting	0	0.00	0	0.00	-
Itching	0	0.00	3	17.65	0.277
Constipation	0	0.00	2	11.76	0.466

**Figure 1:**
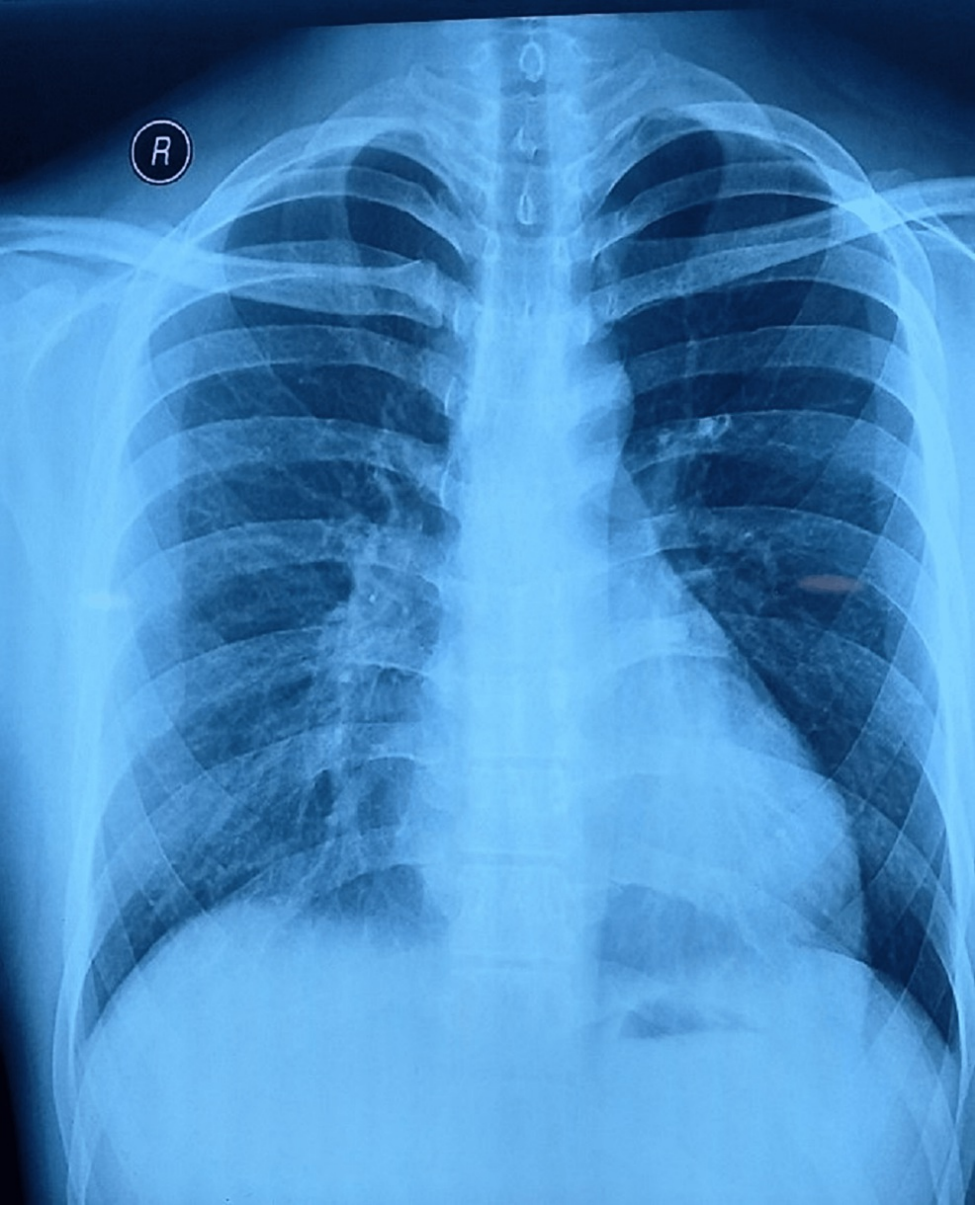
Pre-operative X-ray image of the chest The image depicts the pectus excavatum deformity before the Nuss procedure was done.

**Figure 2:**
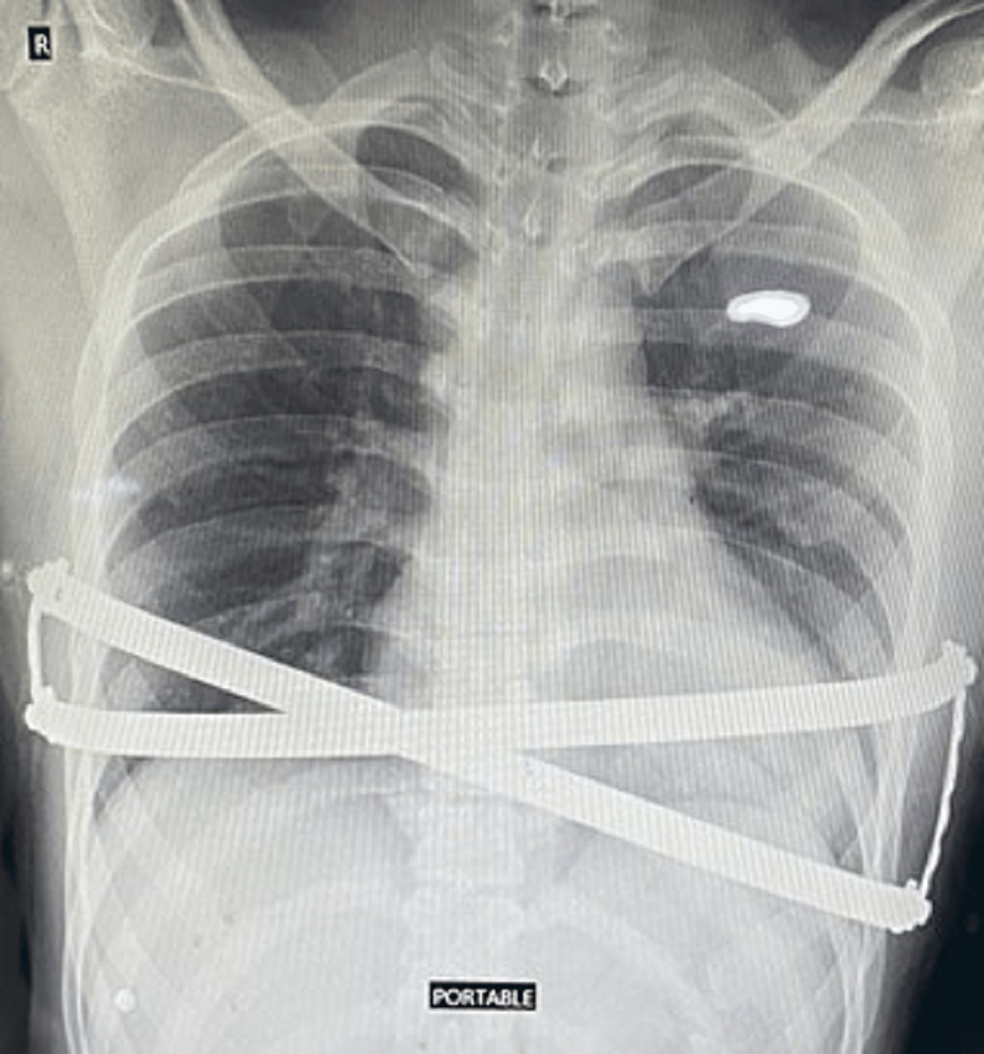
Post-operative X-ray image of the chest X-ray picture of the patient's chest after the Nuss procedure.

Figures 1-2 depict pre-and post-operative chest X-ray images of the pectus excavatum deformity.

## Discussion

The present study indicates that there was no significant difference in the efficacy of post-operative analgesia after the Nuss procedure in pectus excavatum repair between IVPCA and CADD epidural morphine. The pre-operative and post-operative X-ray depictions are given in Figure 1 and Figure 2, respectively. Overall, no significant effect of the type of analgesia was observed on the VAS score, and interaction was not seen between the time of the VAS score assessment and the type of analgesia except at 24 and 72 hours. Similarly, no effect of the type of analgesia was significantly found for the Ramsay Sedation Score (RSS), concluding that both the analgesic regimes produced the same level of sedation and have the same effect. This can be attributed to the fact that opioids were used in low doses in our study and local anesthetics were not included in the epidural. Similar was also observed by other researchers [8,9].

In our study, the mean age, weight, and gender were comparable between groups. The mean height and body mass index significantly differed between groups due to different racial populations (Caucasians, Egyptians, and Africans). Similar findings have been reported by other researchers also [8-10]. Cassady et al. [8] compared continuous thoracic epidural analgesia with bupivacaine- fentanyl combination and PCA with morphine in adolescents undergoing posterior spinal fusion and found them comparable in terms of effectiveness and safety. Similarly in our study, both the groups were comparable for VAS and RSS. Kavanagh et al. [9] carried out a meta-analysis to understand the current procedures and techniques for controlling the post-operative after thoracic surgery. In their study, they compared the usage of various drugs like opioids, NSAIDs, ketamine, and regional analgesia on post-operative pain. They concluded that when thoracic epidural local anesthetics are combined with opioids, they can essentially diminish the post-thoracotomy pain, but there should be considerable monitoring of the possible expected complications and cost-benefit issues. In our study, the VAS score was found to be lower in IVPCA morphine as compared to CADD morphine, but a significant difference was found at 12 hours and 72 hours only. Ramsay sedation scores were also comparable in both groups in our study.

Bloch et al. conducted a study to compare the infusion of tramadol with epidural morphine for post-thoracotomy pain in adults and they finally gave the inference that thoracic epidural analgesia might lead to recovery of respiratory functions at the earliest, but the technique was not found risky [10]. In our study, hospital stay and required rescue analgesia dose after the Nuss procedure were lower in group IVPCA morphine as compared to group CADD morphine, but the duration of hospital stay was significantly reduced in the IVPCA morphine group than in the CADD morphine group.

In our study, the percentages of nausea, vomiting, itching, and constipation were 5.88%, 0.00%, 0.00%, and 0.00%, in Group 1 (IVPCA morphine) and 17.65%, 0.00%, 17.65%, and 11.76%, in Group 2 (CADD morphine), respectively, clearly indicating the safety of both the procedures. The percentage of complications was not significantly different in Group 1 and Group 2. Similarly, Lejus et al. [11] assessed the role of epidural analgesia with bupivacaine and fentanyl among 348 children, and observed effective pain control in 86% of the enrolled patients, with complications that were mild in nature (like nausea, vomiting, pruritus, and urinary retention) in a minor subset of patients. This was in concordance with the study by Rawal N [12] who reported that the life-threatening risk of respiratory depression among patients with IVPCA was approximately 0.9%, which was very less.

The following study provides a vivid description of the role of analgesic morphine given through different routes in alleviating the post-operative pain of pediatric children undergoing surgery for pectus excavatum. There is a paucity of evidence for the two methods used in our study. Hence this study will serve as a prototype for future research in this domain. The limitation of this study is that there was a significant difference in the BMI of the patients in both the groups which was not further analyzed. This might be the foundation of further research adjusting this parameter.

## Conclusions

The Nuss procedure is a minimally invasive procedure for the pectus excavatum or sunken chest, which uses an arch bar for correction. Acute postoperative pain management is crucial for early recovery, better wound healing, less stay in the hospital, and less financial burden to attendants. In this study, we compared two groups, the first one was the intravenous patient-controlled analgesia group and the second group was the computerized ambulatory delivery device-administered analgesia group.

We concluded that the mean VAS score was lower in Group 1 as compared to Group 2, but significantly different at 12 and 72 hours only. The mean hospital stay (days) and requirement of rescue analgesia doses were lower in Group 1 compared to Group 2, but hospital stay (days) was significantly different. Both IVPCA morphine and CADD morphine were effective in controlling post-surgical pain in the Nuss procedure. Both groups had comparable improvement in VAS (except at 12 weeks), hospital stay, and safety. However, IVPCA morphine was better than CADD morphine as it had a better post-operative analgesic effect.
